# Structural Racism as an Environmental Justice Issue: A Multilevel Analysis of the State Racism Index and Environmental Health Risk from Air Toxics

**DOI:** 10.1007/s40615-021-01215-0

**Published:** 2022-01-06

**Authors:** Camila H. Alvarez

**Affiliations:** Department of Sociology, University of California–Merced, 5200 N. Lake Rd., CA 95343 Merced, USA

**Keywords:** Environmental justice, Structural racism, Multilevel modeling, Critical race quantitative methods, Air pollution, Neighborhood effects

## Abstract

Communities of color and poor neighborhoods are disproportionately exposed to more air pollution—a pattern known as environmental injustices. Environmental injustices increase susceptibility to negative health outcomes among residents in affected communities. The structural mechanisms distributing environmental injustices in the USA are understudied. Bridging the literatures on the social determinants of health and environmental justice highlights the importance of the environmental conditions for health inequalities and sheds light on the institutional mechanisms driving environmental health inequalities. Employing a critical quantitative methods approach, we use data from an innovative state racism index to argue that systematic racialized inequalities in areas from housing to employment increase outdoor airborne environmental health risks in neighborhoods. Results of a multilevel analysis in over 65,000 census tracts demonstrate that tracts in states with higher levels of state-level Black–white gaps report greater environmental health risk exposure to outdoor air pollution. The state racism index explains four-to-ten percent of county- and state-level variation in carcinogenic risk and noncarcinogenic respiratory system risks from outdoor air toxics. The findings suggest that the disproportional exposure across communities is tied to systematic inequalities in environmental regulation and other structural elements such as housing and incarceration. Structural racism is an environmental justice issue.

**Supplementary Information:**

The online version contains supplementary material available at 10.1007/s40615-021-01215-0.

“One of the most important indicators of one’s health is one’s street address” [1, p. 2]. -Robert D. Bullard and Beverly Wright

In 2011, air pollution caused an estimated 107,000 premature deaths in the USA—more than traffic accidents and homicides combined [2]. However, these numbers were not equally distributed across the population, but rather reflected the inequalities of US society. A recent PNAS study reported that while not-Latinx, white people are exposed to 17 percent less pollution than they consume, Black and Latinx people are exposed to over 50 percent more pollution than they consume [3]. Communities that are exposed to higher levels of air pollution, a pattern known as environmental injustice, experience serious health consequences. The structural mechanisms driving the distribution of environmental injustices in the USA are understudied. Understanding these injustices in their social context requires recognizing the role that systematic racism plays in creating environmental disparities.

A recent body of research [4, 5] shows that systematic racism contributes to the Black/white gap in health outcomes including infant mortality and cardiovascular diseases. While these studies have made a significant contribution to the literature on racial/ethnic health disparities, one aspect of systematic racism—environmental conditions—remains understudied [6]. On the other hand, a long line environmental justice literature focuses on the environmental conditions to neighborhood-level health outcomes. Environmental justice research [7–10] reveals systematic forms of oppressions such as residential segregation, urban poverty, and mass incarceration contribute to racial/ethnic and socioeconomic environmental disparities. Moreover, research shows these institutional mechanisms put all racial and ethnic groups more at risk for pollution exposure [8]. Exposure to air toxics, such as diesel fumes, particular matter, ethylene oxide, and formaldehyde, has both acute and chronic health consequences, many of which, such as respiratory and cardiovascular problems, are the focus of the health literature [11]. The associations between economic, residential, and environmental injustices highlight the necessity of viewing environmental justice as a “freedom struggle” [12, p. 14]. However, quantitative environmental justice research focusing on the role of systematic racism outside of residential and economic dimensions remains sparse.

Bridging the literatures on the social determinants of health and environmental justice emphasizes both the importance of the environmental conditions for health inequalities and the institutional mechanisms that drive environmental health inequalities. This article draws on the social determinants of health literature by using the state racism index to demonstrate the importance of institutional mechanism in generating disparities in environmental health risks from air pollution. Further, we adopt a critical quantitative methods approach by situating the empirical study within critical race theory. We expand the extant research by focusing on how structural racism influences neighborhood-level environmental health risk from air pollution. We use a cross-sectional multilevel analysis on data from over 65,000 census tracts. Results reveal tracts in states with a higher state-level Black-white gaps have a higher level of estimated cancer risk and noncancer respiratory system risks from outdoor air toxics for all racial and ethnic groups. This suggests that systematic inequalities in environmental regulation and other aspects of the social structure such as housing and incarceration may lead to worse air pollution. Thus, the findings emphasize the importance of the environmental justice literature for expanding research in other fields such as public health and the sociology of race and ethnicity.

## Health Disparities: The Role of Structural Racism

Researchers who focus on the social determinants of health use eco-social theory to explain disparities in outcomes by race, class, and gender within social systems [13, 14]. Eco-social theory moves beyond individualistic explanations for health outcomes, asserting that health is embodied through the “societal and ecological context” [13, p. 214]. One significant aspect of the social system that leads to health disparities is racism, defined as an ideology of racial oppression at various levels, from interpersonal to institutions, that has real material consequences [15].

Racism operates in both micro- and macro-level settings. Interpersonal racism entails person-to-person interactions and can be either deliberate or unintentional. In contrast, structural racism occurs via institutions and policies. Structural racism theory is aligned with both Bonilla-Silva’s [16] “racialized social systems,” which he defined as the process of social, political, economic, and ideological dimensions becoming institutionalized to form racialized outcomes, and Feagin’s systematic racism theory, which describes “the foundational, large-scale and inescapable hierarchical system of US racial oppression devised and maintained by whites and directed at people of colour” [17, p. 936]. A central aspect of systematic racism is the disproportionate allocation of material resources leading to real material consequences including environmental and health disparities [15, 16].

A recent systematic review of studies in the fields of public health and structural racism found that most research on the social determinants of health research emphasized discrimination measures [6]. The review stressed the importance of examining housing, criminal justice, and political system as forms of structural racism. Recent advances have improved the measurement and evaluation of structural racism, thus paving the way for a more nuanced understanding health disparities [18–22]. In one of the first of these studies, Lukachko et al. [4] examined the influence of state-level structural racism using a novel measure: Black/white ratios of political participation, employment and job status, educational attainment, and judicial treatment. The results shows that Black individuals living in states with a high level of structural racism had higher rates of myocardial infarction, while white individuals living in these states reported lower rates of myocardial infarction. In a follow-up study, Wallace [5] also used Black/white ratios, in this case ratios of income, education, and incarceration, and found that Black infant mortality was significantly higher in states with a higher level of structural racism. Finally, a recent piece by Mesic et al. [23] expanded the use of the state racism index to demonstrate its effect on Black-white inequality in police shooting. Most of this important work has focused on individual-level outcomes; however, systematic racism can affect larger-scale outcomes such as neighborhood conditions.

Health research focused on place, specifically neighborhood effects, has expanded in recent years, and health researchers agree that local environments are important for health outcomes [24]. Indeed, Castle et al. called on researchers to emphasize the neighborhood or “socioecological framework” in studies of health outcomes [6, p. 33]. Place-based health research focuses on how both structural inequalities and neighborhood context affect health [25]. Analyzing the way that systematic racism distributes unequal health risks via differential exposure to air pollution entails moving from the individual as the unit of analysis to a higher ecological level such as neighborhoods [26] because environmental inequalities occur at the neighborhood level. For example, the development of an industrial facility that emits pollutants—or the creation of an accessible green area that has environmental benefits—impacts not just one family but an entire neighborhood. A review of the research on neighborhood effects on health found that the majority of these studies focused on two outcomes: obesity and mental health [24]. In addition, most studies focused on socioeconomic variables at the neighborhood level, such as rates of owner-occupied housing, unemployment, and educational attainment. In a notable exception from this pattern, Smiley [27] examined air pollution at the neighborhood level and its corresponding health outcomes in southern states; the results showed that a neighborhood’s levels of residential segregation and air pollution exposure were significant predictors of asthma prevalence rates. In the current study, we advance the research on neighborhood effects on health by focusing on how structural racism distributes health outcomes via differential levels of environmental pollution. In addition to the research on the structural determinants of health, the environmental justice research provides an important foundation for this analysis.

## Environmental Justice: Bringing environmental conditions into the analysis of health risks

Environmental justice is the right to a clean environment and workplace. The environmental justice movement focuses on eradicating environmental inequalities, defined as toxic hazards being disproportionally placed in communities of color and among poor residents [12, 28, 29]. Importantly, these hazards are not produced by the individuals living in affected communities, but rather by neighboring industrial facilities, transportation systems, or military sites [ 70. , 30, 31]. Individuals who live in areas with a higher level of toxics in the environments, whether in the air, in the water, or on land, have a higher risk of developing health problems.

One of the first environmental justice reports, which was published by the United Church of Christ Commission for Racial Justice, revealed environmental inequalities in the form of landfill facilities being disproportionately located in Black and Latinx communities. Rev. Ben Chavis, then-director of the commission, defined environmental racism as the extension of racial discrimination to environmental policies, a lack of enforcement, and targeting communities of color for toxic facilities and thus exposing them to more risk [32]. Environmental racism, which is a form of institutional racism, can consist of “any policy, practice, or directive that differentially affects or disadvantages (whether intended or unintended) individuals, groups, or communities based on race or color” [33, p. 497]. Chavis [32] noted that the exclusion of marginalized peoples from decision-making processes contributes to the production of environmental inequalities [32]. Activism and research in the field of environmental racism have facilitated legal victories and informed the development of policy recommendations [34]. Because pollution happens at larger ecological levels, it is reasonable to argue that environmental conditions at the neighborhood level affect the health of everyone in the locale. In other words environmental racism and “environmental inequality can reduce environmental quality” [35, p. 29].

A key goal of environmental justice research is identifying the structural mechanisms that distribute environmental inequalities. For example, a long line of research on air pollution reveals Black and Latinx communities are exposed to higher rates of industrial air toxics [36, 37]. Moreover, the research has demonstrated the joint role of overlapping social dimensions including race/ethnicity, nationality, gendered family structures, and socioeconomic status in producing cumulative environmental inequality [26, 36–38]. Most of the environmental justice research considers individual-level socioeconomic characteristics such as race, income, educational attainment, and employment rates as possible mechanisms generating environmental inequalities [36, 37, 39, 40]. By comparison, there is less quantitative environmental justice research examining the role of institutional racism on distributing environmental inequalities.

Systematic racism is engendered through municipal and federal policies, and this contributes to the formation of environmental injustices. For example, the Home Owners’ Loan Corporation Act, which was enacted in 1933 as part of the New Deal, set the stage for redlining by enacting a process in which neighborhoods were assigned a rating from A-D (“A” being the best rating) [28]. The rating system was used to assess mortgage risk of neighborhoods, however racist assumptions about social demographics were included [41]. Thus, newly developed suburbs with a majority of white residents were given a rating of A, while industrial neighborhoods with a majority of Black residents (or residents of color) were assigned ratings of B-D. The impact of neighborhoods being redlined, or given a lower score, persists even today in the form of decreased tree canopy covering and greater outdoor air pollution [8, 42]. In addition, housing corporations and federal housing programs enacted policies that supported white flight—the process of white residents moving away from industrial hubs and urban cores to the suburbs [28]. This shift ultimately led to the decentralization of cities and the rise of suburbanization and thus reinforced environmental injustices including air pollution disparities [43].

State racism indices from the social determinants of health literature gauge structural inequalities through a combination of Black-white ratios in housing, incarceration, educational attainment, economic status, and employment. In contrast, environmental justice researchers have examined these factors *separately* to assess environmental inequalities. For example, scholars have used several indices of residential segregation and found that racial residential segregation predicted greater exposure to pollution for all racial groups, but African-Americans had greater exposure as compared to white residents [8, 44]. Recent work examines the carceral state as an environmental justice issue in part because “prisons and jails in the US are institutions where people of colour are overrepresented and are frequently built adjacent to or even on top of toxic waste sites, are inundated with air and/or water contamination, and are sources of hazardous waste generation.” [10, p. 2]. Previous environmental justice literature combines the structural forms of educational attainment, economic status, and employment to examine the *degree of social and political capital*—where neighborhoods with less social and political capital are more likely to have locally unwanted land uses such as manufacturing sites or major highways. This type of work uses an economic deprivation index encompassing rates of employment, residential tenure status, educational attainment, and gendered-family structure to assess the role of poverty and deindustrialization on the location of Superfund sites and air pollution disparities [7, 37]. Taken together, we can see that greater structural inequalities compromises health conditions via environmental pollution exposure. Drawing on findings from research on structural racism, the social determinants of health, and environmental justice, we employ a critical quantitative approach to analyze environmental inequality.

## Critical Race Quantitative Studies: A QuantCrit Approach to Environmental Inequality

Grounding methodological approaches within a critical perspective is essential, especially when assessing structural racism and environmental justice. Critical race theory, which was first developed by legal scholars, emphasizes race as a social construction and asserts that race is reinforced through institutions and policies. The field focuses on understanding the roles of race and racism in social systems and working to eradicate racial inequalities and racism. While the original literature centered on the legal system, scholars have since applied critical race theory to other fields, such as the sociology of race and ethnicity, as well as epistemological and ontological frames, including the use of quantitative methods to understand racialized disparities [45, 46].

Researchers have historically used social statistics to justify and reinforce racist assumptions and ideologies [46, 47]. In the nineteenth and twentieth centuries, prominent social statisticians developed the field to strengthen the pseudoscience of eugenics. As Zuberi noted, “[e]ugenic ideas were at the heart of the development of statistical logic” (35). The rationale and motivation behind the development of statistical tools such as those used to measure difference and probability (in the absence of evidence supporting casual inference) was to support white supremacy [47]. In a notable exception to this pattern; W.E.B. Du Bois used sociological methods, including statistics, from a critical perspective to identify the racial disparities that emerged due to systematic racism [48, 49]. Unless researchers confront the legacy of white supremacy within the field of social statistics, it will persist. Thus, QuantCrit theoretically situates the use of quantitative methods within a critical race theory perspective.

Gillborn, Warmington, and Demack [45] outlined five tenets of the QuantCrit approach: 1) racism is not easily quantifiable because it is a complex system of oppression and is entrenched in many aspects of society; 2) numbers are not independent from social and political bias and should be examined for their role in supporting analyses that enforce white racial interests; 3) the categories used in quantitative analyses are not inherent and should be critically examined; 4) data itself is not sufficient and critical analyses should also recognize the voice and on-the-ground experiences of marginalized groups; and 5) quantitative analyses are not essential but can help be used in the support of social justice.

While most quantitative environmental justice research comes from a racial justice perspective, it is important to explicitly state the role of structural racism in distributing environmental inequalities. As stated within the tenets of QuantCrit, numbers are not neutral and therefore should be contextualized within a critical theoretical framework. We center the current analysis of environmental inequalities within the systematic racism framework to emphasize the structural mechanisms that place Black, Indigenous, and other people of color, as well as poor people, in neighborhoods with a higher exposure to environmental toxins. Moreover, the consequences of structural racism on air pollution compromises the environmental quality for everyone [50].

## Research Question

Important research demonstrates Black, Latinx, Asian, and Indigenous communities are exposed to greater levels of air pollution, however less research has focused on systematic patterning of health risk via environmental pollution due to institutional racism. Previous research demonstrates air pollution disparities is linked to levels of residential segregation and economic disadvantage [7, 8, 37, 44, 51]. Recent work connects mass incarceration as a significant system of oppression of environmental inequality [10]. We argue the spatial embodiment of structural racism is tied to the placement of pollution emitting sites (e.g., manufacturing sites or major transportation structures) and environmental-friendly amenities (e.g., parks or bike trails), and their placement have larger neighborhood effects that makes the environment worse for everyone. Here we use the state racism index, measured as an aggregate of Black-white gaps, from the social determinants of health literature to examine the distribution of structural racism on estimated cancer risk and noncancer respiratory system risk from air pollution. Our research question is: Do neighborhoods located in states with higher state racism index report greater environmental health risk from outdoor air toxics?

## Unit of Analysis

The unit of analysis for the study is census tracts. The sample includes over 65,000 census tracts across the contiguous USA and in Alaska and Hawaii. Census tracts are proxies for neighborhoods and are commonly used in research on how neighborhood characteristics influence health [24]. Tracts are a sensible unit of analysis because a tract is the smallest unit available in many data sets. Data for the environmental health risk and demographic control variables are measured at the census tract, while the state racism index is measured at the state level. Metropolitan status is reported at the county level, and EPA region is at the state level.

## Dependent Variable: Estimated cancer risk and noncancer respiratory system risk from outdoor air toxics

Data on air toxics were drawn from the National Air Toxics Assessment (NATA) published by the Environmental Protection Agency (EPA), which reports the concentrations of the air toxics listed in the Clean Air Act and Clean Water Act and estimates environmental health risks from air toxics exposure [52]. NATA data are based on rigorous procedures for assessing airborne emissions and modeling health consequences. We matched the 2011 NATA estimates to each census tract. The focal analyses use two tract-level standardized measures: estimated cancer risk due to exposure to air toxics in a 70-year lifespan per million people and noncancer hazard risk quotient for respiratory system (see Supplementary Fig. 1–2 for national maps). The noncancer hazard risk quotient is the ratio of estimated exposure to the level of exposure for noncancer health risk. Thus, higher levels indicate greater noncancer health risks. We decided to use the estimated cancer risk variable for its easy interpretability and it has been deployed in several environmental justice research [31, 37]. However, one limitation of the cancer measure is the cancer threshold of air toxics can be conservative due to political pressures from industry. To address this challenge, we included the noncancer hazard quotient of respiratory system risk because most adverse health problems from air pollution impact the respiratory system. Table 1 presents the descriptive statistics.

**Table 1:** Descriptive statistics

	**Level**	**Mean**	**SD**	**Median**	**Min**	**Max**	**N**
Estimated cancer risk from air toxics	tract	40.045	12.501	39.571	10.742	826.309	72,347
Respiratory system noncancer hazard risk quotient	tract	1.870	1.009	1.701	0.184	40.782	69,205
State racism index	state	46.94	8.96	44.98	21.64	67.98	50
Segregation scale	state	62.70	7.39	63.81	46.00	73.21	50
Incarceration scale	state	36.57	24.19	29.82	0.00	100.00	50
Education scale	state	44.27	20.45	43.31	0.00	100.00	50
Economic scale	state	41.48	16.09	41.10	8.80	74.94	50
Employment scale	state	49.71	13.47	48.46	8.83	80.25	50
Black (%)	tract	13.33	21.87	3.69	0.00	100.00	72,347
Latinx (%)	tract	15.29	20.85	6.21	0.00	100.00	72,347
Indigenous (%)	tract	0.81	4.61	0.24	0.00	100.00	72,347
Asian and Pacific Islanders (%)	tract	4.49	8.59	1.51	0.00	100.00	72,347
Median household income (in $10,000s)	tract	5.76	2.81	5.14	0.38	24.95	70,098
Female-headed household (%)	tract	13.67	8.11	11.56	0.00	100.00	72,242
Renters (%)	tract	35.63	22.49	29.99	0.00	100.00	72,242
Metro (binary)	county	0.37	0.48	0	0	1	3,142

## State Racism Index

We use the state racism index published in Mesic et al. [23] to operationalize structural racism. The state racism index is the average of five scales: residential segregation and Black-white ratios in incarceration, educational attainment, economic status, and employment. The state racism index is a standardized measure that can be used to compare across states (a major benefit). However, the standardization can cost real-world meaning of the measure. Let it be clear, that the state racism index should be interpreted as a gauge of Black-white inequalities of various societal dimensions. Higher scores indicate greater systematic Black-white gaps. Below we outline the specific data and measures used to calculate each scale. Unless stated otherwise, data were downloaded from the National Historical Geographical Information System [53]. We conclude by discussing the calculation of the state racism index.

Data on *residential segregation* are from the 2010 U.S. Census. This dimension includes two measures: dissimilarity and isolation. The dissimilarity index measures the percent of Black residents who would have to move to obtain equal percentages of Black and white residents in each area. The isolation index measures the geographic isolation of racial groups and assesses how likely a Black resident comes into contact with another Black resident. Both measures are in 0–100 scales. The residential segregation scale is the average of the dissimilarity and isolation scores.

Data on *incarceration* are drawn from the Prison Policy Institute’s statistics for 2010 [54]. This dimension consists of the ratio of the proportion of Black people who are incarcerated to the proportion of white people who are incarcerated. This ratio was normalized to 0–100 scale^1^ and represents the incarceration scale.

*Educational attainment* data are from the American Community Survey (ACS) 1-year estimates for 2010. The state of Wyoming was missing educational attainment data for the 1-year 2010 estimates; thus, we used the ACS 3-year 2008–2010 estimate. The educational attainment dimension is the ratio of the proportion of Black people without a college degree to the proportion of white people without a college degree. This ratio was normalized to 0–100 scale and represents the educational attainment scale.

The data on *economic status* come from the ACS 1-year estimates for 2010. Montana and Wyoming are missing data so we used the 5-year estimates for 2006–2010. The economic status dimension includes three ratios: poverty, median household income, and renters. The poverty measure is the ratio of the proportion of Black residents living under the poverty line to the proportion of white residents living under the poverty line. The median household income measure is the ratio of the median household income for Black residents to the median household income for white residents. The renters measure is the ratio of the proportion of Black households with tenure of renter to the proportion of white households with tenure of renter. Each ratio was normalized to 0–100 scale and economic status scale is the average of the normalized ratios of poverty, median household income, and renters.

The *employment* data comes from the ACS 1-year for 2010. For states with missing data, we used the 5-year estimates for 2006–2010.^2^ The employment dimension includes two ratios: the ratio of the proportion of Black people who are not participating in the labor force to the proportion of white people who are not participating in the labor force and the ratio of the proportion of Black people who are unemployed to the proportion of white people who are unemployed. Every ratio was normalized to a 0–100 scale. The employment scale is the average of the normalized ratios of people not participating in the labor force and unemployment.

**Fig. 1:**
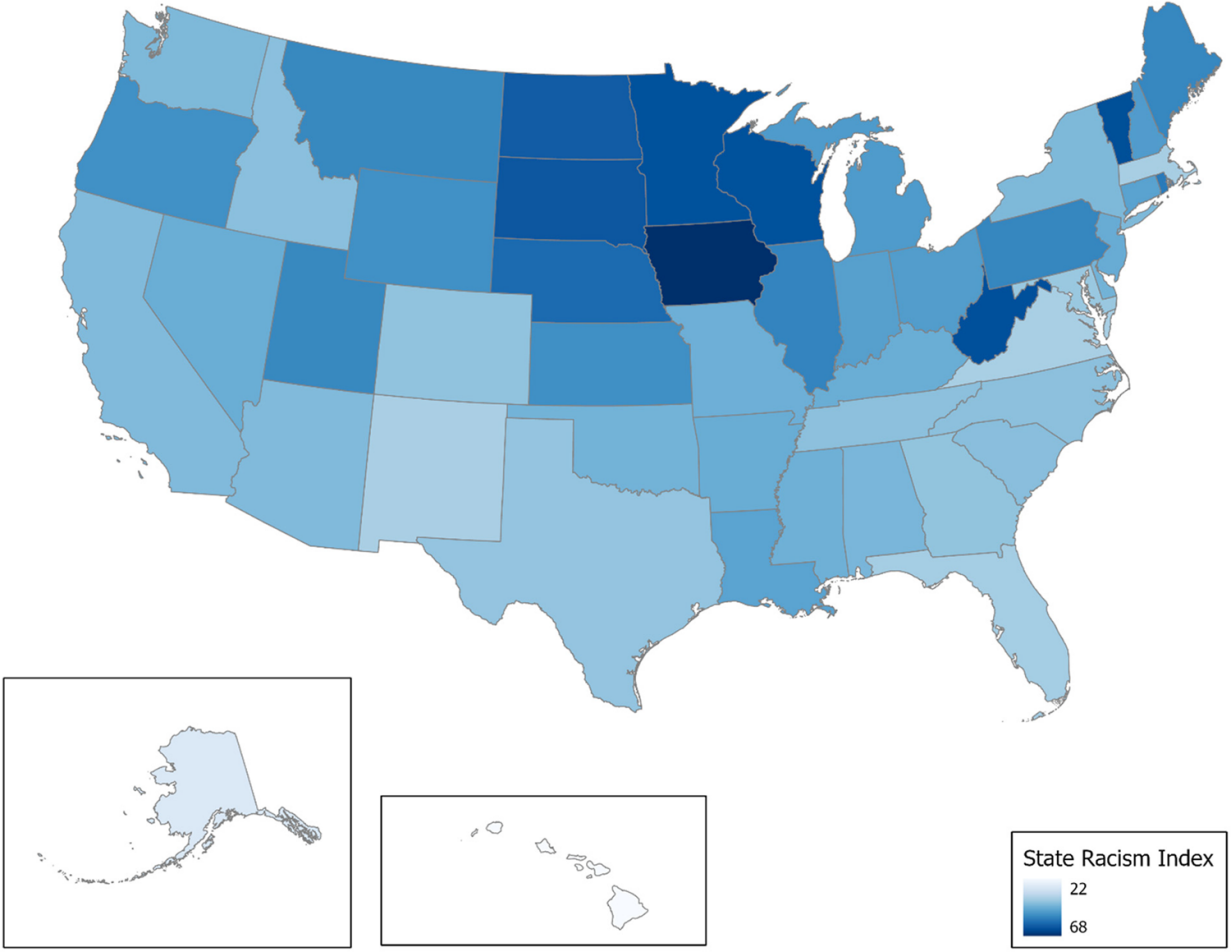
State racism index

The *state racism index* is the average of segregation, incarceration, educational attainment, economic status, and employment scales. The way to interpret the index is higher values of the state racism index indicate greater Black-white gaps. Figure 1 maps the state racism values and for the exact values see Supplementary Table 1.

## Control Variables

Control variables were used to assess whether environmental inequalities could be explained by other factors. Previous environmental justice as well as research on structural racism demonstrate the importance of racial and ethnic communities and median household income [23, 36, 51]. We controlled for tract-level percent Black, percent Latinx, percent Asian and Pacific Islander, and percent Indigenous. Earlier work on environmental inequality demonstrates the importance of gendered family household and housing tenure, so we controlled for tract-level percent female-headed household and percent renters [7, 26, 37, 55]. Data on race/ethnicity, female-headed household, and renter status were from the U.S. Census 2010. Median household income data came from the ACS 2010–2014 wave. Multiple studies [56, 57] illustrate tract-level data from the ACS can be unreliable, so we removed tracts with coefficient of variation values greater than 0.4. Finally, we follow previous work [36, 39, 58] and control for geographical factors. We include USDA Economic Research Service Rural–Urban continuum data on metropolitan status because urban areas also tend to be areas of concentrated pollution [59]. Moreover, EPA regions is important to control for because environmental regulation happens at that level. EPA region 2 was chosen as the reference category because it had the largest average estimated cancer risk. Models with control variables reduced the sample size to 70,098 census tracts for estimated cancer risk and 67,031 census tracts for noncancer respiratory system risk. To assess the multicollinearity, we examined the variation inflation factor (VIF) for the fully saturated model of estimated cancer risk and noncancer respiratory system risk. Each independent variable reported a VIF value lower than 5, thus indicating multicollinearity is not an issue [60].

## Analytic Approach: Multilevel Modeling

Ordinary least squares regression assumes that observations are independent of each other. However, census tract data from the same county or state are likely to be similar due to geographic proximity. In fact, previous research demonstrates a large amount of clustering happening at the county level [61]. To account for this sameness or dependence, we use a multilevel modeling approach with three levels: census tracts nested within counties nested within states [62]. The logic of multilevel modeling is to start with the null model and then develop the model by adding fixed effects or independent variables. In the current analysis we use a random intercept model:

$${y}_{ijk}=\beta {\delta }_{ijk}+{v}_{0k}+{\mu }_{0jk}+{e}_{0ijk}$$

$${v}_{0k}\sim N\left(0, {\sigma }_{v}^{2}\right)$$

$${\mu }_{0jk}\sim N\left(0, {\sigma }_{\mu }^{2}\right)$$

$${e}_{0k}\sim N(0, {\sigma }_{e}^{2})$$

where ${y}_{ijk}$ is the estimated cancer risk or noncancer respiratory system risk from outdoor air toxics (i.e., the outcome variable) for census tract *i* in county *j* in state *k*; ${\delta }_{ijk}$ is the vector of the intercept and fixed effects (i.e., state racism index or percent of Black residents) for tract i, county *j,* and state *j*; and $\beta$ is the parameter coefficient. The random effects are ${\sigma }_{v}^{2}$ at the state level, ${\sigma }_{\mu }^{2}$ at the county level, and ${\sigma }_{e}^{2}$ at the tract level. All random effects are assumed to be normally distributed. Generally, the intercept represents the overall average of environmental health risk when all other factors are held at zero across all counties and states. The fixed effects represent the direction and magnitude of environmental health risk for each independent variables across all counties and states. The random effects capture the error or residual difference between tracts, counties, and states of the estimated models.

To evaluate the differences between models, we use the variance partition coefficient (VPC) and the proportional change of variance (PCV). The VPC is the percentage of the total variance explained by the higher levels (in this case the county and state levels). The VPC is reported for each model and is calculated as:

$$VPC=\frac{{\sigma }_{\mu }^{2}+{\sigma }_{v}^{2}}{{\sigma }_{e}^{2}+{\sigma }_{\mu }^{2}+{\sigma }_{v}^{2}} * 100\%$$

The PCV is calculated in between models and represents the proportional difference in higher-level variances [63, 64]. The PCV is calculated between hypothetical Models 1 and 2 as:

$$PCV=\frac{\left({\sigma }_{Model 1:\mu }^{2}+{\sigma }_{Model 1:v}^{2}\right)-({\sigma }_{Model 2:\mu }^{2}+{\sigma }_{Model 2:v}^{2})}{\left({\sigma }_{Model 1:\mu }^{2}+{\sigma }_{Model 1:v}^{2}\right)}$$

## Results

Census tracts have an estimated cancer risk from air toxics as low as 10 diagnoses per million people across a 70-year lifespan and as high as 826 diagnoses—nearly 83 times higher (Table 1). The average of noncancer hazard risk quotient for respiratory system is 1.870 and ranges from 0.184 to 40.782. Scores on the state racism index range from 26.96 to 64.72, revealing variation in Black-white gaps across segregation, incarceration, educational attainment, economic status, and employment across states.

## Assessing the association between the state racism index and environmental health risk from outdoor air pollution

Tables 2–3 present the results of the multilevel regression models where Table 2 reports results for the dependent variable of estimated cancer risk and Table 3 demonstrates the results for noncancer health risk to the respiratory system. Tracts in states with a higher state racism index report a significantly higher environmental health risk in cancer risk and noncancer respiratory system risks. In Model B of Tables 2–3, the coefficient for the state racism index is significant and positive, meaning that an increase in the state racism index (a state-level predictor) corresponds to an increase in environmental health risk from outdoor air pollution in estimated cancer risk and noncancer health risk for respiratory system. The variation test statistics (explained above) provide further support for this hypothesis. In Model A of Table 2 the VPC is 69.56 percent, meaning that almost 70 percent of the variance in estimated cancer risk across the tract level is explained at the county and state levels. Moreover, the VPC of the null model in Table 3 shows about 47% of the variation of noncancer respiratory risk is explained at the county and state levels. The results for estimated cancer risk and noncancer respiratory risk show the importance of using a multilevel approach (rather than ordinary least-squares regression) in this analysis.

**Table 2:** Multilevel Regression Results for Estimated Cancer Risk from Outdoor Air Toxics

	**Model A**			**Model B**			**Model C**		
	**Estimate**	**SE**	**P**	**Estimate**	**SE**	**P**	**Estimate**	**SE**	**P**
FIXED EFFECTS									
Intercept	31.215	1.167	0.000	14.621	5.109	0.004	12.678	4.943	0.010
State racism index				0.357	0.108	0.001	0.192	0.070	0.006
Black (%)							0.004	0.003	0.158
Latinx (%)							0.048	0.002	0.000
Indigenous (%)							-0.045	0.008	0.000
API (%)							0.059	0.004	0.000
Median household income (in $10,000s)							0.065	0.016	0.000
Female-headed household (%)							-0.005	0.007	0.502
Renters (%)							0.155	0.002	0.000
Metro (binary)							5.067	0.268	0.000
EPA Regions									
1							-2.487	3.383	0.462
2 (reference)									
3							3.593	3.296	0.276
4							11.804	3.108	0.000
5							-0.607	3.157	0.848
6							11.283	3.367	0.001
7							0.821	3.374	0.808
8							-6.941	3.396	0.041
9							0.460	3.661	0.900
10							-0.887	3.725	0.812
RANDOM EFFECTS									
State	65.910	13.482		53.901	11.034		13.617	2.935	
County	53.255	1.506		53.251	1.506		39.690	1.143	
Tract	52.154	0.280		52.154	0.280		40.397	0.220	
VPC	69.56%			67.26%			56.89%		
PCV (from null model)				10.08%					
N	72,347			72,347			70,098

**Table 3:** Multilevel Regression Results for Noncancer Respiratory System Risk from Outdoor Air Toxics

	**Model A**			**Model B**			**Model C**		
	**Estimate**	**SE**	**P**	**Estimate**	**SE**	**P**	**Estimate**	**SE**	**P**
FIXED EFFECTS									
Intercept	1.230	0.051	0.000	0.595	0.233	0.011	0.310	0.258	0.229
State racism index				0.014	0.005	0.005	0.008	0.004	0.033
Black (%)							0.001	0.000	0.000
Latinx (%)							0.005	0.000	0.000
Indigenous (%)							-0.005	0.001	0.000
API (%)							0.003	0.000	0.000
Median household income (in $10,000s)							0.004	0.001	0.002
Female-headed household (%)							-0.001	0.001	0.114
Renters (%)							0.011	0.000	0.000
Metro (binary)							0.379	0.020	0.000
EPA Regions									
1							-0.240	0.177	0.177
2 (reference)									
3							0.008	0.170	0.964
4							0.235	0.160	0.141
5							-0.096	0.162	0.555
6							0.280	0.173	0.106
7							-0.099	0.174	0.570
8							-0.360	0.176	0.041
9							-0.112	0.192	0.560
10							0.270	0.194	0.164
RANDOM EFFECTS									
State	0.119	0.026		0.103	0.022		0.034	0.008	
County	0.246	0.008		0.246	0.008		0.178	0.006	
Tract	0.406	0.002		0.406	0.002		0.334	0.002	
VPC	47.35%			46.24%			38.78%		
PCV (from null model)				4.37%					
N	69,205			69,205			67,031		

The VPC reported in Table 2 falls slightly to 67.26 percent in Model B, which is expected as the fixed effect of the state racism index explains a portion of the variance. For Model B in Table 3, the VPC decreases to forty-six percent. The PCV which represents the percentage difference between the county- and state-level variances between Model A and B in Table 2 is 10.08 percent. In other words, the fixed effect of the state racism index explains about 10 percent of the county and state levels of estimated cancer risk. Table 3 reports four percent of variation in noncancer respiratory health risk from the county and state level is explained by the state racism index.

**Fig. 2:**
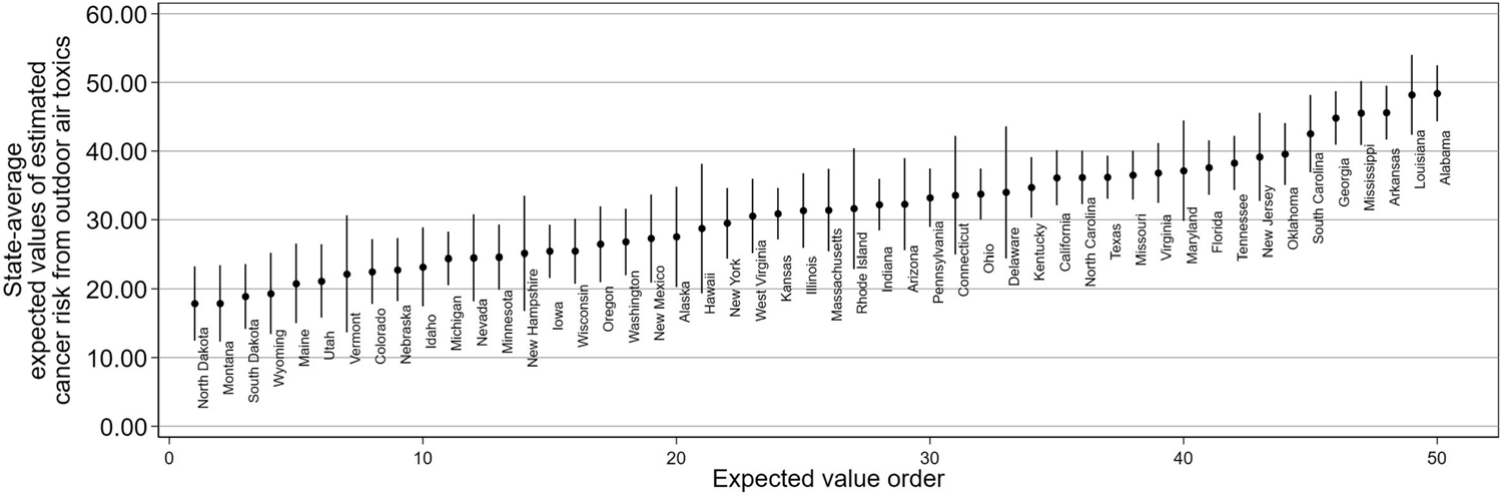
State-average Predicted Values for Estimated Cancer Risk from Outdoor Air Toxics

**Fig. 3:**
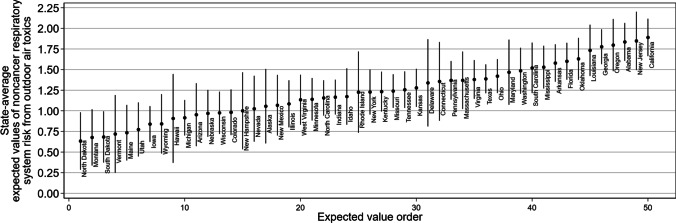
State-average Predicted Values for Noncancer Respiratory System Risk from Outdoor Air Toxics

Figures 2–3 illustrate between-state differences for each environmental health risk variable by plotting the state-level averages of the expected values of environmental health risk from Model B reported in Tables 2–3. Higher expected values mean higher environmental health risks. For estimated cancer risk, Fig. 2 reports most of the states with the highest expected values (Alabama, Louisiana, Arkansas, Mississippi, and Georgia) are in the South. With the exception of Maine, most of the states with the lowest expected values (North Dakota, Montana, South Dakota, Wyoming, and Maine) are in the mountain west and northern plains. The census tracts in Alabama, the state with the highest expected value of environmental health risk (48.40), have an estimated cancer risk from air toxics almost 2.71 times higher than the tracts in North Dakota, the state with the lowest expected value of environmental health risk (17.87).

Figure 3 is a caterpillar plot of noncancer respiratory system risk and the states reporting the highest expected values of risk are in the west, south, and northeast region (California, New Jersey, Alabama, Oregon, and Georgia). The states estimated to have lower expected values of risk are located in mountain west, northern plains, and northeast (North Dakota, Montana, South Dakota, Vermont, Maine, and Utah). The state-level expected value for noncancer respiratory system risk for California (1.89) is almost 2.98 times higher than North Dakota (0.63).

**Fig. 4:**
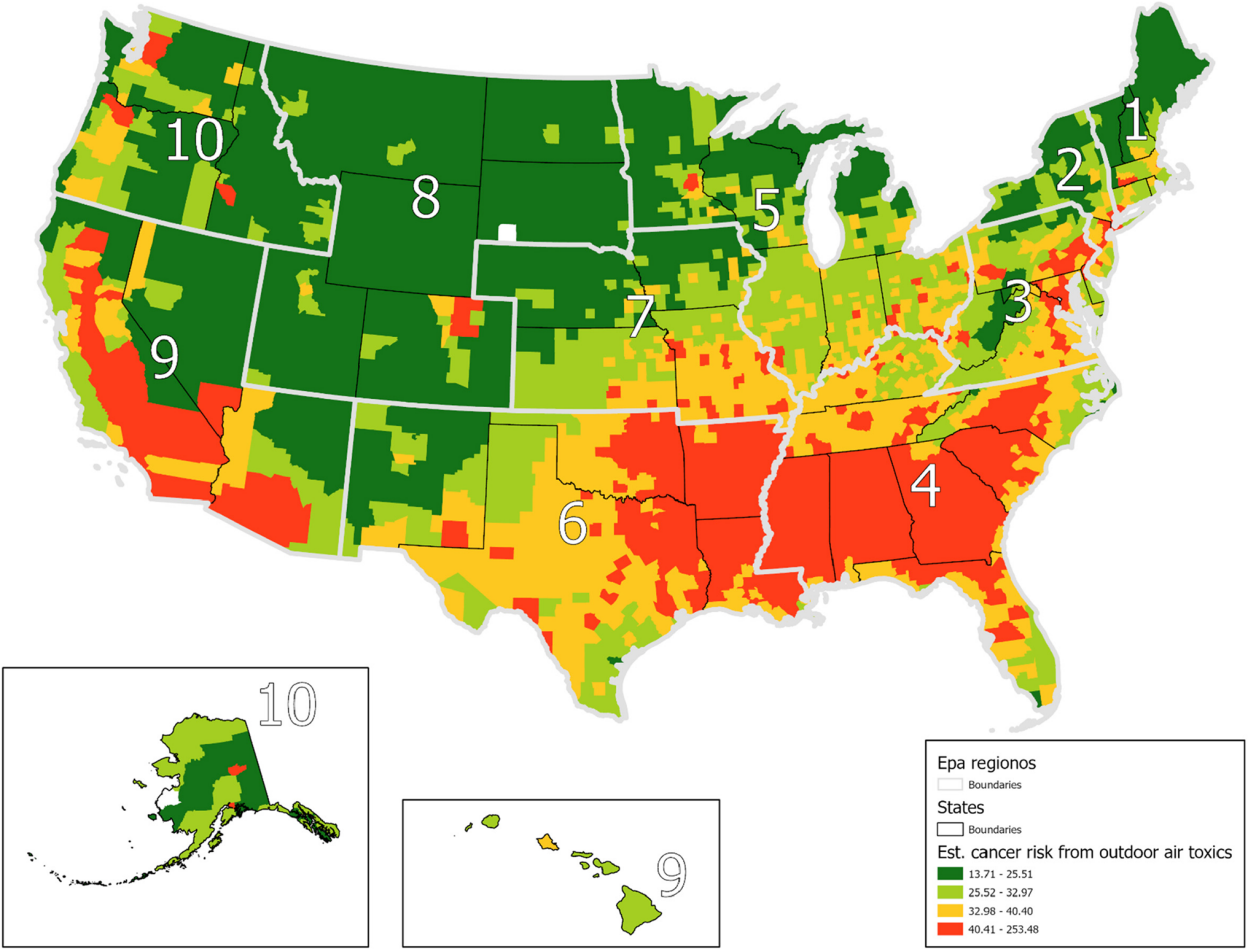
County-average Predicted Values for Estimated Cancer Risk from Outdoor Air Toxics with EPA Regions. *Notes:* Predicted values of estimated cancer risk due to exposure to air toxics in a 70-year lifespan per million people at the county level from Model B from Table 2

**Fig. 5:**
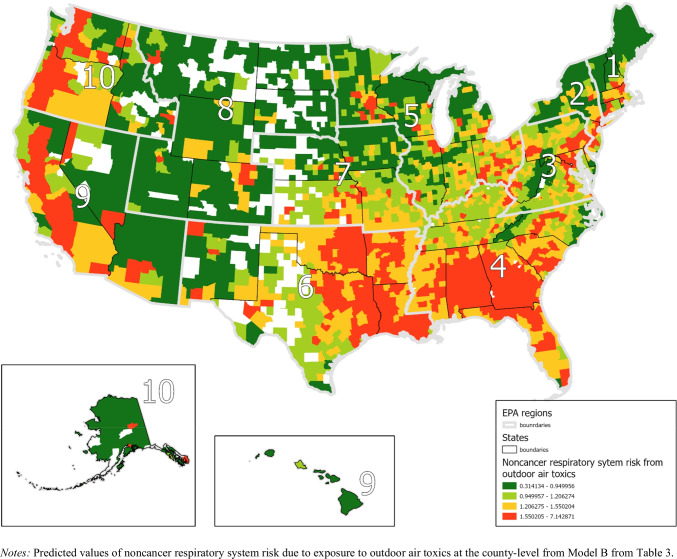
County-average Predicted Values for Noncancer Respiratory System Risk from Outdoor Air Toxics with EPA Regions

To further demonstrate the regional variation in the results, Figs. 4–5 present quantile maps of the expected values at the county level based on the results from Model B from Tables 2–3. Figure 4 demonstrates areas with the highest estimated cancer risk from outdoor air toxics are located primarily in the southeast, parts of the southwest, and California. The expected values map for noncancer respiratory system risk (Fig. 5) shows the highest values in the southeast, west, and northeast regions.

## Robustness of the association between the state racism index and environmental health risk

The results of Model C reported in Tables 2–3 offer evidence that the association between the state racism index and environmental health risk is robust to the inclusion of a set of pertinent control variables. Model C in Tables 2–3 are random intercept models that include control variables commonly used in environmental justice literature (percent of racial/ethnic residents, median household income, percent of female-headed household, and percent of renters). Moreover, the models include place control variables: metropolitan status and EPA regions.

Among tract- and county-level predictors, Model C in Table 2 reports positive and significant coefficients for percent of Latinx residents, percent of Asian and Pacific Islander residents, median household income, percent of renter residents, and metro status; meaning increases in these predictors leads to increase estimated cancer risk from outdoor air toxics. Percent of Indigenous residents reports a negative and significant coefficient. In addition, the results of Model C from Table 2 show that EPA regions 4 and 6, which are in the south-central and eastern portions of the country, have significantly higher environmental health risk than EPA region 2 (the reference region). In contrast, EPA region 8, which is in the mountain northwest, has a significantly lower environmental health risk than EPA region 2. The VPC falls to 57 percent indicating that the variables added in Model C account for a significant amount of the explained variance.

Model C in Table 3 reports positive and significant coefficients for percent of Black residents, percent of Latinx residents, percent of Asian and Pacific Island residents, median household income, percent of renter residents, and metropolitan status thus indicating as these variables increases there is a corresponding increase in noncancer respiratory system risk. Percent of Indigenous residents is negative and significant suggesting Indigenous communities may experience lower noncancer respiratory system risk. EPA region 8 reports significantly lower noncancer respiratory system risk as compared to EPA region 2. The VPC of Model C in Table 3 falls to about 39%.

The association between the state racism index and estimated cancer risk and noncancer respiratory system risk remain significant even when racial/ethnic composition, socioeconomic status indicators, urbanicity, and EPA region are controlled. This pattern of results reveals that the presence of structural racism on environmental health risk are not *entirely* explained by regional variation.

## Discussion and Conclusion

We found significant variation in estimated cancer risk and noncancer respiratory system risks from outdoor air pollution across the USA (see Table 1). The findings indicate that while much of this variation is explained by differences in sociodemographic characteristics and systematic indicators (Tables 2–3), neighborhoods in states with greater systematic racial inequalities (high residential segregation and significant Black-white gaps in incarceration, educational attainment, economic status, and employment) have a significantly higher estimated cancer risk and noncancer respiratory system risk from exposure to air toxics. Independent of other factors, we found additional estimated cancer risk from outdoor air pollution for Latinx and Asian and Pacific Islander communities. For noncancer respiratory system risk, we found Black, Latinx, and Asian and Pacific Islander communities were exposed to greater environmental health risk, independent of each other. In addition, there are regional differences that reflect various economies (see Figs. 4–5). For example, estimated cancer risk from air pollution is high in the southern USA. Despite the aggregate regional differences, state-level racism manifests to be an important indicator. This is reflected by the PCV—indicating nearly a tenth of the variation of estimated cancer risk and four percent of the variation of noncancer respiratory system risk—between counties and states can be explained by the state racism index. Further, the effect of the state racism index on cancer risk and noncancer respiratory system health risk is robust to the inclusion of percent of racial/ethnic minority residents, socioeconomic status, metropolitan status, and EPA regions. Thus, the results highlight the importance of systematic racism to issues of environmental justice.

Using a critical quantitative methods approach, we situate the findings within critical race theory. The findings demonstrate the importance of identifying neighborhood-level environmental conditions (i.e., outdoor air pollution exposure) for understanding systematic racism. We found that the greater systematic racism of a state is linked to greater levels of outdoor air pollution in all neighborhoods. This aligns with previous environmental inequality work demonstrating environmental inequalities affect mostly communities of color and poor neighborhoods, however they also affect other communities [65]. Systematic forces such as environmental regulation and enforcement often drive the placement of major pollution contributors, such as major transportation infrastructures, industrial facilities, or military installations [ 70. , 30, 31]. The oversight of environmental issues is interconnected to the discriminatory enforcement of housing and civil right laws [1]. Zoning and land-use decisions are often made by the state and influenced by industries that ignore those who experience the environmental and health consequences of their actions [66, 67]. These consequences influence the environmental conditions at the neighborhood level by poisoning the air, water, and land. Thus, the mechanisms that drive social inequalities are interlinked with greater pollution and in turn affect everyone in the locale [50].

Weaken regulation oversight and democratic participation in social and political power can make communities more susceptible to the *negative externalities* of pollution [65]. This reflects previous work highlighting the importance of equitable power distribution—including support for procedural justice, tax oversight, and social welfare programs—in political and social arenas [68]. Systematic racism can erode public goods and equitable power distribution which in turn may compromise environmental quality [50]. For example, redlined neighborhoods continue to have less green space (a type of environmental amenity) than those that were not restricted [42]. These policies have generated significant environmental inequality—research on the socioenvironmental transformation of places has shown that the proportion of communities of color residing in and near lands used for industrial purposes increased in the twentieth century. Our results demonstrate state racism is linked to worse outdoor air pollution for all neighborhoods. This suggests policies addressing to mitigate systematic racism, including those related to democratic practices such as voting rights, oversight, and social programs, may improve environmental justice.

No study is without limitations, and the current analysis entails five noteworthy constraints. First, while we are confident in our use of the state racism index (borrowed from the health literature), prior studies have used other measures such as quantiles of the various Black/white ratios. We used an aggregate index comprised of various normalized scales of Black-white gaps. Critical race theory scholars emphasize that racism is a complex system that must be evaluated through multiple lens including law, experience, and statistics. Moreover, the USA is a systematically racist country. Studies like the one presented here use the state racism metric to assess *the degree* of structural racism between states. Certain states may score higher than others, but all states are characterized by structural racism. Everyone, including the authors, have a lot of work to do. Future studies should further consider the presence, complexity, and nuances of systematic racism. Second, the analysis uses cross-sectional data, and it is not built to estimate causal effects. We situate the analysis within theoretical framework of structural racism in order to draw casual hypothesis from the statistical associations. Future research using panel analysis would test the robust causality of the findings. Third, EPA data is widely used in the environmental justice literature; future research should analyze air pollution concentrations and integrate individual-level health outcomes in a multilevel approach. Fourth, because neighborhood characteristics can affect people differently based on their social position [69], researchers should examine whether there are racialized differences in the effects of state racism on environmental exposure. Analyses of individual-level health data have revealed such differences. Finally, we focused on systematic racism, however, given the interconnectedness of systems of oppression it would be fruitful to adopt an intersectional approach, examining overlapping systems of power such as structural racism, patriarchy, and capitalism.

We utilized an environmental justice perspective to argue that structural racism worsens environmental health risks and to demonstrate the importance of environmental conditions for health disparities. As the USA and the world continue to grapple with immense environmental, health, and social inequality problems, researchers must evaluate these interconnected crises to situate them within a structural context and inform potential solutions.

## Supplementary Information

Below is the link to the electronic supplementary material.

Supplementary file1 (DOCX 1057 KB)

## Data Availability

Not applicable.

## Footnotes

1 Each ratio was normalized to a 0–100 scale:

$${x}^{^{\prime}}=\frac{\mathrm{x}-{\mathrm{x}}_{minimum}}{{\mathrm{x}}_{maximum}-{x}_{minimum}}*100$$

2 Idaho, Montana, North Dakota, South Dakota, Vermont, and Wyoming are missing data.
