# Supplementary material for: Structural Racism as an Environmental Justice Issue: A Multilevel Analysis of the State Racism Index and Environmental Health Risk from Air Toxics
Source: J Racial Ethn Health Disparities. 2022 Jan 6;10(1):244–58. doi: 10.1007/s40615-021-01215-0 (PMC9810559; doi:10.1007/s40615-021-01215-0)

**Supplementary Table 1: State racism index by state**

| **State** | **Segregation Index** | **Incarceration Index** | **Educational Attainment Index** | **Economic Status Index** | **Employment Index** | **State Racism Index** |
| --- | --- | --- | --- | --- | --- | --- |
| Alabama | 51 | 9 | 46 | 57 | 52 | 43 |
| Alaska | 70 | 10 | 30 | 14 | 17 | 28 |
| Arizona | 66 | 18 | 66 | 21 | 41 | 42 |
| Arkansas | 59 | 17 | 56 | 44 | 50 | 45 |
| California | 67 | 28 | 43 | 26 | 47 | 42 |
| Colorado | 72 | 31 | 10 | 30 | 60 | 40 |
| Connecticut | 65 | 71 | 0 | 53 | 48 | 47 |
| Delaware | 52 | 27 | 43 | 51 | 46 | 44 |
| Florida | 56 | 19 | 40 | 36 | 38 | 38 |
| Georgia | 50 | 6 | 40 | 59 | 47 | 40 |
| Hawaii | 70 | 0 | 20 | 9 | 9 | 22 |
| Idaho | 67 | 24 | 57 | 29 | 29 | 41 |
| Illinois | 55 | 56 | 28 | 57 | 68 | 53 |
| Indiana | 62 | 28 | 53 | 38 | 57 | 48 |
| Iowa | 71 | 85 | 51 | 67 | 66 | 68 |
| Kansas | 65 | 50 | 34 | 41 | 64 | 51 |
| Kentucky | 63 | 27 | 62 | 32 | 41 | 45 |
| Louisiana | 49 | 16 | 41 | 73 | 57 | 47 |
| Maine | 72 | 39 | 53 | 49 | 50 | 52 |
| Maryland | 52 | 23 | 19 | 56 | 47 | 39 |
| Massachusetts | 65 | 26 | 8 | 41 | 47 | 38 |
| Michigan | 56 | 35 | 43 | 50 | 60 | 49 |
| Minnesota | 69 | 76 | 29 | 71 | 65 | 62 |
| Mississippi | 46 | 0 | 46 | 75 | 55 | 44 |
| Missouri | 58 | 24 | 43 | 40 | 57 | 45 |
| Montana | 68 | 67 | 81 | 15 | 30 | 52 |
| Nebraska | 67 | 52 | 34 | 54 | 80 | 57 |
| Nevada | 63 | 16 | 65 | 34 | 46 | 45 |
| New Hampshire | 70 | 45 | 61 | 34 | 33 | 48 |
| New Jersey | 59 | 53 | 19 | 48 | 46 | 45 |
| New Mexico | 65 | 5 | 72 | 11 | 34 | 37 |
| New York | 61 | 42 | 19 | 41 | 50 | 43 |
| North Carolina | 55 | 22 | 34 | 47 | 47 | 41 |
| North Dakota | 72 | 46 | 77 | 46 | 59 | 60 |
| Ohio | 59 | 32 | 43 | 49 | 57 | 48 |
| Oklahoma | 63 | 20 | 58 | 34 | 44 | 44 |
| Oregon | 71 | 38 | 65 | 35 | 45 | 51 |
| Pennsylvania | 59 | 64 | 34 | 45 | 60 | 53 |
| Rhode Island | 71 | 63 | 27 | 60 | 51 | 55 |
| South Carolina | 50 | 20 | 26 | 60 | 50 | 41 |
| South Dakota | 73 | 71 | 53 | 29 | 80 | 61 |
| Tennessee | 56 | 16 | 54 | 35 | 44 | 41 |
| Texas | 61 | 10 | 52 | 30 | 47 | 40 |
| Utah | 68 | 45 | 61 | 33 | 54 | 52 |
| Vermont | 68 | 82 | 100 | 17 | 46 | 63 |
| Virginia | 54 | 29 | 9 | 46 | 50 | 37 |
| Washington | 70 | 33 | 38 | 31 | 40 | 43 |
| West Virginia | 71 | 100 | 72 | 20 | 49 | 62 |
| Wisconsin | 62 | 77 | 34 | 60 | 77 | 62 |
| Wyoming | 70 | 33 | 59 | 44 | 47 | 51 |

**Supplementary Figure 1: County-level estimated cancer risk from air toxics**

**
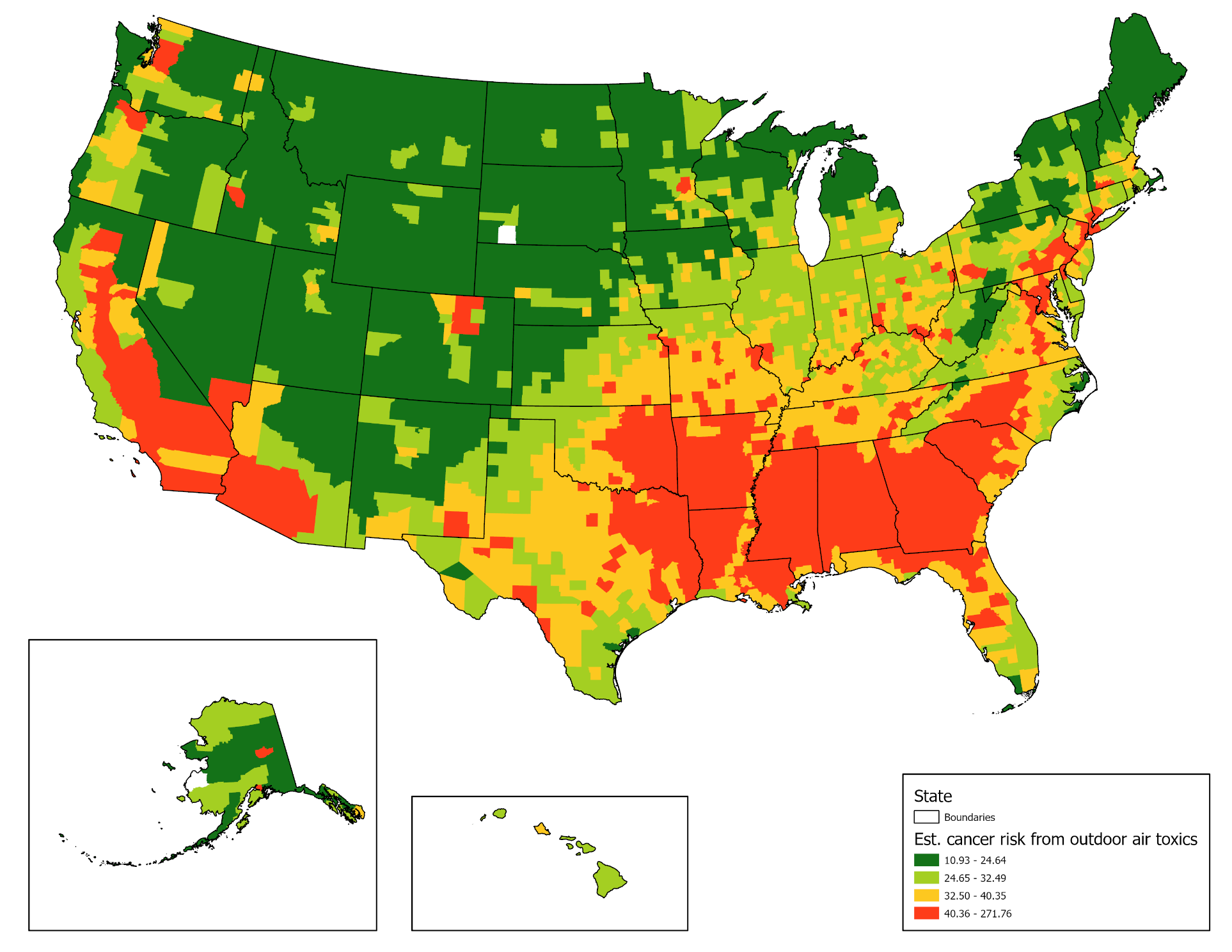
**

**Supplementary Figure 2: County-level noncancer respiratory system risk from air toxics**


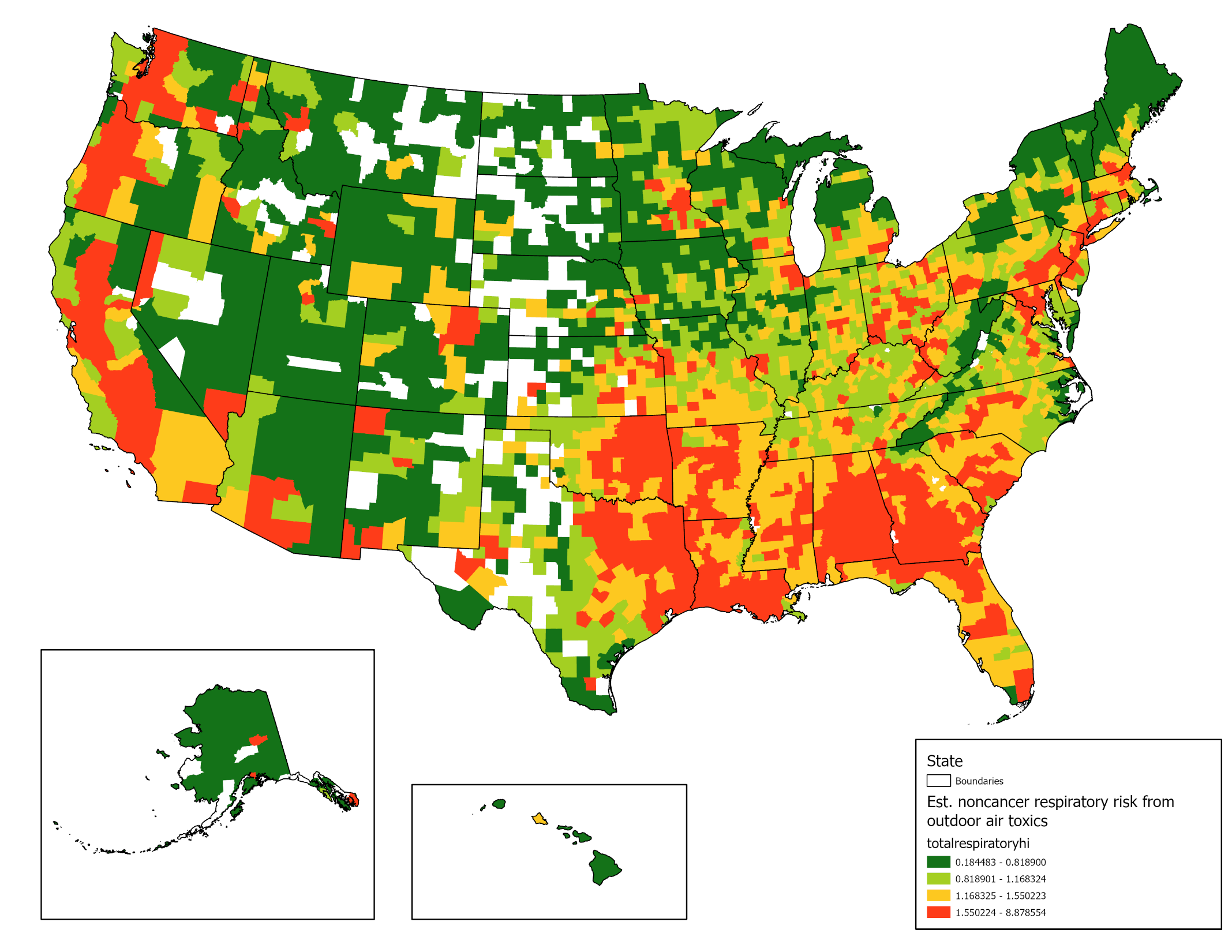

Supplement: Supplementary file 1 — Supplementary file1 (DOCX 1057 KB) [file 40615_2021_1215_MOESM1_ESM.docx]
